# Does growth in the outdoors stay in the outdoors? The impact of an extended residential and outdoor learning experience on student motivation, engagement and 21st century capabilities

**DOI:** 10.3389/fpsyg.2023.1102610

**Published:** 2023-03-06

**Authors:** Jeff Mann, Tonia Gray, Son Truong

**Affiliations:** ^1^School of Education, Western Sydney University, Penrith, NSW, Australia; ^2^School of Health and Human Performance, Dalhousie University, Halifax, NS, Canada

**Keywords:** student engagement, student motivation, 21st Century skills, outdoor learning, outdoor education

## Abstract

**Introduction:**

Student motivation and engagement underpin educational success, and recent research has found they are lowest in middle high school, especially for boys. At the same time, education systems are recognizing that academic performance is necessary but not sufficient to prepare young people for the adult world, and so-called “21st Century skills” (communication, collaboration, critical thinking, and creativity) have been suggested as critical capabilities across all employment sectors in the future. The Glengarry program is a 6-month residential and outdoor learning experience for Year 9 (14–15 years old) boys at an Australian independent school, The Scots College (TSC) Glengarry. Intentionally located during the lowest point of engagement in their adolescent student journey, the Residential and Outdoor Education experience was hypothesized to boost their motivation and engagement and develop 21st Century skills.

**Methods:**

The Glengarry program involves students living in a boarding-style community for 20 weeks away from their families, participating in classes across all regular school subjects at a bush campus, and undertaking increasingly challenging outdoor education trips each week. The study aimed to measure how these factors transferred into students’ traditional school environment after their Glengarry experience. Year 9 was split into two cohorts who both participated in the study: one of which completed the Glengarry program in the first half of 2019, and the other during the second half of the year.

**Results:**

Self-reported quantitative and qualitative data supported the hypothesis that the Glengarry program did indeed, boost student motivation and 21st Century skills. While gains in 21st Century skills endured over the next 8–10 months, motivation and some engagement factors decreased upon return to the traditional school environment. Students described key factors in the Glengarry program which facilitated their development, including: an intense residential environment necessitating social growth, a closer connection with teachers in both school and community life, and an appreciation of learning in the natural environment. Recommendations are made for future research to strengthen these findings, and for how these mediating factors could be incorporated into the regular school environment.

## Introduction

While academic grades are the most easily measured metric of educational success, there are important antecedent factors which contribute to students’ achievement, and a range of outcomes which describe their holistic development. One such factor is students’ sense of engagement, or connection to their learning and the school community. Engagement recognizes that the student is not just an intellectual vessel waiting to be filled, but also needs a positive emotional climate and supportive web of peer and teacher relationships in order for effective and lasting learning to occur. Although educational learning outcomes were previously defined through the narrow lens of academic achievement, there is increasing recognition that young people require a broad set of capabilities to thrive in contemporary society. These proficiencies have been variously defined as “21st Century skills,” in an attempt to categorize the human-centric proficiencies thought to be applicable to every profession and industry. The current study seeks to explore the potential of an extended residential and outdoor school program for enhancing student engagement and building 21st Century capabilities, and how these factors endure on return to the traditional school environment.

### Student engagement

Student engagement has been described as a multifaceted construct with a myriad of definitions in the literature (e.g., Appleton et al., 2008). At its simplest, engagement is about a significant connection, such as when a couple marks a new level of commitment in their relationship by becoming “engaged” (Yazzie-Mintz, 2006). Students’ engagement with their learning has often been defined with behavioral, affective and cognitive dimensions (Fredricks et al., 2004). Behavioral engagement relates to participation in schooling activities; affective engagement describes emotional ties to school created by reactions to peers, teachers and school authorities, and; cognitive engagement relates to a student’s willingness to exert effort in order to understand ideas and master skills. An alternate but similar framework includes social (including sense of belonging, participation in voluntary school activities, and positive friendships), academic (attendance and absence frequency) and intellectual (emotional and cognitive investment using higher order thinking skills) dimensions of student engagement (Willms et al., 2009).

The terms “motivation” and “engagement” are both attached to distinct sets of literature, and there is also an intersection between the two sets. Maehr and Meyer (1997) noted that motivational theories had begun to inform educational practice, and Self Determination Theory (Ryan and Deci, 2000) is an example of a prominent psychological conceptualization of motivation which has been applied extensively in educational research (e.g., White et al., 2021). Appleton et al. (2008) argued that motivation is essential to understanding engagement, however engagement is a construct in its own right. In Australian research, Martin (2005) initially described motivation as “students’ energy and drive to engage, learn, work effectively, and achieve to their potential at school” and engagement as “the behaviours that follow from this energy and drive” (p. 180), but then used both terms as one phrase in later writing (Martin, 2008a,b, 2009; Martin et al., 2016). A model which synthesizes these theories proposes that student motivation answers the question about the reasons *why* students do what they do at school, as suggested by Appleton et al. (2008). Engagement is concerned with *how* students “do” school (Martin, 2005)—how they cognitively apply learning strategies, how they emotionally feel about being in their school community, and how they behaviorally participate in various school activities (Fredricks et al., 2004). Educational outcomes are *what* results from this cognitive, affective and behavioral engagement with school.

Engagement with the social and learning environment at school has been proposed as an essential condition for effective student development (Abbott-Chapman et al., 2014; Gallup, 2014; Griffiths and Webber, 2017; Mann, 2018), and has been correlated with various educational outcomes. Early research studies conducted in the late 20th Century suggested that students reporting higher engagement tend to earn higher grades, perform better on tests, and drop out at lower rates, and also that lower levels of engagement place students at risk for negative outcomes such as lack of attendance, disruptive classroom behavior, and leaving school early (Klem and Connell, 2004). Appleton et al. (2008) similarly reviewed a range of research studies which established evidence for the connection between engagement, achievement, and school behavior across levels of economic and social advantage and disadvantage. In a study of 11,800 French-Canadian high school students, a global measure of engagement reliably predicted early high school dropout, and behavioral engagement (attendance, completion of classwork and homework, participation in school activities) contributed to this accuracy while affective and cognitive engagement dimensions did not (Archambault et al., 2009). A study of over 78,000 United States students in 160 schools across eight states showed that a 1% increase in emotional engagement (defined as enthusiasm for school) was associated with a 6% increase in reading and an 8% increase in mathematics achievement, while controlled for socio-economic status (Gallup, 2014). A student-voice survey of 272,000 Canadian middle and high school students found that the 40% of students who considered they had high skills and were similarly challenged in their learning (i.e., were intellectually engaged) were less likely to report experiencing anxiety and depression (Tramonte and Willms, 2012). Public school students in NSW who reported higher behavioral engagement (i.e., attentiveness in class and abiding by school rules) in Year 7 were 7 months ahead in reading performance in Year 9 (McCarthy and McCourt, 2017). Analysis of nationally representative data on Australian 12–13 year old students showed that cognitive and affective engagement was a mediating factor of socio-economic status on academic achievement (Tomaszewski et al., 2020). An Australian longitudinal study of 6,600 students aged between 9 and 15 years old found that each unit on a six point affective engagement scale (i.e., enjoyment of school) was associated with a 10% higher chance of completing a post-school qualification, as well as higher status occupations 20 years later (Abbott-Chapman et al., 2014). A recent study in the United States found that cognitive and behavioral engagement levels of senior high school students predicted both college enrolment and persistence (Fraysier et al., 2020). Recognizing its multidimensional nature, student engagement has been shown to significantly contribute to in-school and post-school outcomes.

Although research has shown the importance of student engagement, recent indicators suggest that many Australian students are disengaged, particularly in mid-high school and especially boys (Goss et al., 2017; Griffiths and Webber, 2017). A Western Australian longitudinal study by Angus et al. (2009) found that 40% of primary and lower secondary students showed consistent unproductive behaviors in class, and that these disengaged students were one to 2 years behind their peers in academic performance. Furthermore, only a quarter of these unproductive students were actively disruptive, while over half were described as compliant but disengaged. A review for the Western Australian government similarly indicated that 25% of 15 year-old Australian students thought that school had not prepared them for adult life, and 22% felt that they did not belong at school (Hancock and Zubrick, 2015). In a measurement of Australian student engagement over the academic lifespan including 23,000 participants, Martin (2009) found that elementary students had the highest level of engagement, followed by university students and that high school students were least engaged. Fifteen year-old students’ sense of belonging in school was analyzed across the 38 countries which participated in the 2003 and 2012 rounds of the Program for International Student Assessment (PISA) testing, and Australia had the fifth highest decrease in affective engagement during this period (Organisation for Economic Cooperation Development, 2013). Within Australia, a survey of 79,000 New South Wales students across Year 7–12 showed that sense of belonging, positive relations with teachers and perceptions of teacher expectations were at their lowest in middle high school, which has been coined “the Year 9 dip” (Willms, 2015). This age-related low point in school belonging and aspirations to finish Year 12 was also seen in the same survey 2 years later, and an analysis of gender effects revealed that boys were less likely to aspire to finish high school, aim to go to university, exhibit positive behavior at school, and have positive relationships with their teachers (Griffiths and Webber, 2017). International data similarly shows that boys tend to be less engaged with school in most developed countries (Organisation for Economic Cooperation Development, 2015), and a review of research on gender and engagement reported that boys generally show lower levels of engagement, and behavioral engagement particularly (Lietaert et al., 2014). When considering gender differences in student engagement, it should be acknowledged that these trends do not describe all boys and all girls as homogenous groups (Martino, 2008), and that boys and girls may exhibit engagement or disengagement differently (Griffiths and Webber, 2017).

Willms (2015) argued that “student voice” is one of the best ways to measure engagement, as it directly accesses the social and affective aspects of engagement. Student surveys are an effective method to capture student voice, as they can access a large number of students quickly and cost-effectively. On the other hand biased responses and accuracy of self-perception are drawbacks to student surveys, however these disadvantages can be mediated by triangulating data from student qualitative data and other sources in the school community, such as teachers or parents (McCourt and Griffiths, 2016).

### 21st Century skills

As we progress through the 21st Century, there is a growing recognition by researchers and governments that school outcomes need to be broader than just academic grades, and should also include meta-cognitive and socio-emotional skills (Lamb et al., 2017; OECD, 2019). These capabilities have always been important, of course, but recent challenges like automation of lower order tasks, globalization of the workforce, rising mental health issues, climate change and mass migration make them particularly relevant to this century (Lambert, 2017). The emerging economic, educational and social ripples of the COVID-19 pandemic can be added to this list of 21st Century challenges (Pendergast, 2022; Mann et al., 2022a; Adams and Gray, 2023). Anderson and Jefferson (2018) suggested that “the skills which equip young people to engage with the world of work are the same skills that will help them live life to the full as 21st Century citizens” (p. 14).

Similar to student engagement research, there are many models of 21st Century skills with varying lists of capabilities (Trilling, 2009; Fullan and Langworthy, 2013; World Economic Forum, 2015; Fadel, 2016; Anderson and Jefferson, 2018; OECD, 2019). Four factors are common to most frameworks: the cognitive skills of creative and critical thinking, and the social skills of communication and collaboration. Creativity refers to divergent thinking which sees problems from new perspectives and encourages playing with possibilities; critical thinking challenges assumptions, asks key questions, and adapts knowledge to new applications; communication includes identification of verbal and non-verbal messaging, conveying meaning and purpose, and enabling agency, and; collaboration incorporates the offering of ideas, shaping of these ideas in an emotionally safe context, and co-constructing new solutions (Anderson and Jefferson, 2018).

Fadel (2016) suggested these higher order skills are essential for students to deeply engage with academic content, and for demonstrating their understanding of disciplinary knowledge. 21st Century skills can be seen in the recent development of a national Australian Curriculum as the General Capabilities of critical and creative thinking, and personal and social capabilities (Australian Curriculum Assessment and Reporting Authority, 2018), as well as featuring in various Australian state curricula (Lambert, 2017). While many educational systems across the world recognize the importance of 21st Century capabilities, there are few examples of how these should be operationalized in terms of specific teaching, learning and assessment strategies (Lamb et al., 2017). In the Australian context, the Australian Council for Educational Research has recently developed a pilot resource for assessing creative thinking, collaboration and critical thinking in a project-based learning context (Scoular et al., 2020).

Research into 21st Century capabilities is only just emerging, and Lamb et al. (2017) described a dense web of overlapping theories in this area. Although some theorists seem to describe 21st Century skills as the panacea for modern education, in fact research is yet to determine whether the skills are specific to a disciplinary domain (e.g., mathematical critical thinking) and the extent to which they may be transferable between domains (Lamb et al., 2017). The connection between 21st Century skills and traditional academic performance is also yet to be rigorously explored, however 21st Century capabilities can stand as useful educational outcomes in their own right rather than simply being valued as mediators of academic progress.

### Learning outdoors

The outdoor “*in situ*” environment has been the setting for learning across most of human history (Nicol and Waite, 2020), and the indoor classroom only became the “normal” place of learning with the advent of mass schooling in the 19th Century (Mann et al., 2021). Contemporary outdoor learning has been classified into two forms (Mann et al., 2022a): outdoor adventure education (OAE), which utilizes challenge and perceived risk to create cognitive dissonance (Priest and Gass, 2005), and curricular-based learning outside the classroom (LOTC) which incorporates student-led experiential learning principles (Beames et al., 2012). Outdoor learning contexts include: school gardens, school playgrounds, local parks and forests, field trips, residential camps and wilderness trips (Mann et al., 2022b). An ample body of research has demonstrated the benefits of both OAE (Gray, 1997; Hattie et al., 1997; McLeod and Allen-Craig, 2007) and LOTC (Becker et al., 2017; Mygind et al., 2019; Miller et al., 2021) across a range of socio-emotional and wellbeing outcomes such as: self-concept, interpersonal skills, mental and emotional health, environmental knowledge and attitudes, learning dispositions and academic progress (Mann et al., 2022b).

School attendance has been considered as a metric of behavioral engagement, and two small studies in the United Kingdom showed improved attendance in vulnerable primary (McCree et al., 2018) and junior secondary (Price, 2015) student groups who undertook student-led and adventurous activities in natural settings. School motivation was directly measured in a larger Danish study of 28 primary classes from 18 schools, which found that regular subject-based LOTC across 1 year improved students’ self-reported motivation (Bølling et al., 2018), however there has been little other research into the potential for outdoor learning to boost student motivation and engagement. There is good evidence for OAE and LOTC developing the 21st Century skills of communication and collaboration, and for nature exposure generally to benefit critical and creative thinking, however there is a paucity of research into the potential for outdoor learning experiences to develop these cognitive 21st Century skills (Mann et al., 2022a).

### Summary

**Figure 1:**
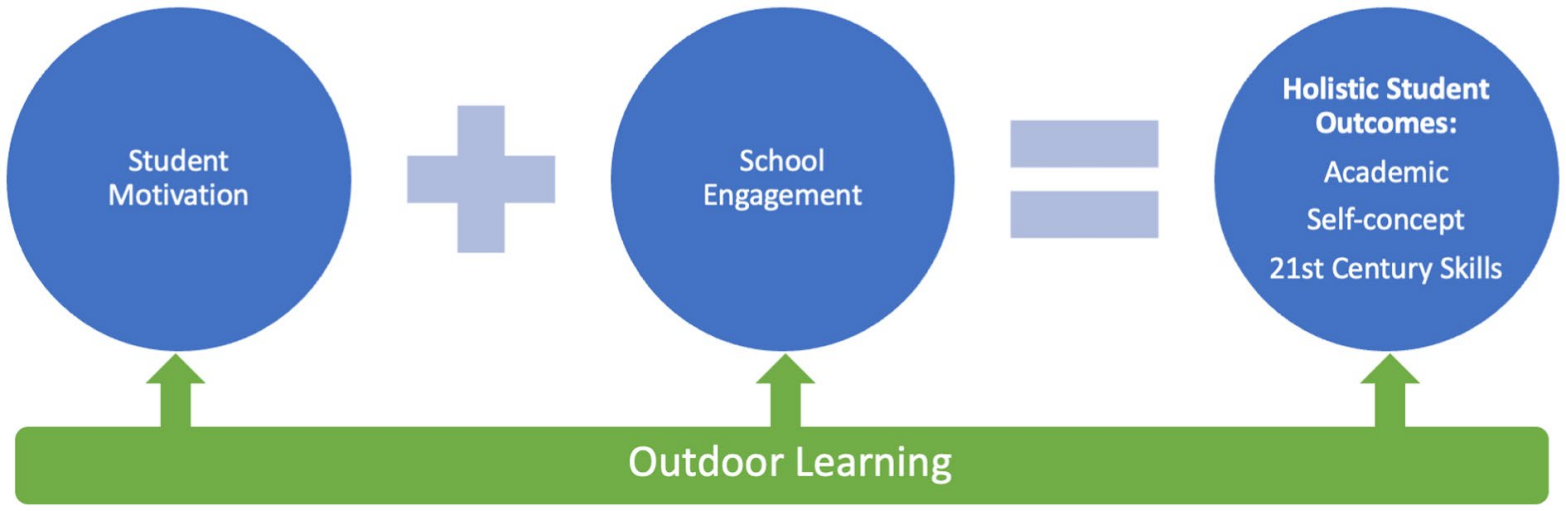
Hypothesized boosting effect of outdoor learning on student motivation, school engagement, and holistic student outcomes.

Student motivation and engagement are essential pre-requisites for school learning, and 21st Century skills are becoming widely regarded as important educational outcomes along with academic knowledge. Learning in natural outdoor environments could be a prime context for both engaging students in learning and growing 21st Century capabilities (Figure 1), however there has been little research to date connecting these areas.

Assuming outdoor learning experiences could enhance student engagement and 21st Century skills, students also need to translate these gains back into their normal school context for enduring growth. Although the last stage of the established framework for experiential learning is active experimentation in a new environment (Kolb, 1984), transfer of learning on OAE programs back into the school environment has typically been a challenge (Sibthorp et al., 2011) and there has been little research into the mechanisms of this learning transfer (Bobilya et al., 2015).

While the research described above has shown strong evidence for OAE developing various self-identity factors, the Glengarry program provides an opportunity to examine the effect of OAE and LOTC on the educationally-focused constructs of student engagement and 21st Century skills. The current study aims firstly to ascertain whether the Glengarry residential outdoor learning program influences students’ sense of engagement and 21st Century capabilities, and secondly whether any gains are transferred into their traditional school environment following completion of the program. The following research questions are proposed:

1. Does an extended residential and outdoor program increase student engagement and 21st Century skills?
2. Are changes in student engagement and 21st Century skills enduring once the students return to their normal school environment?
3. What do students perceive are the contributing factors to hypothesized changes in these areas, and their variation over time?

## Methods

### Background

The Scots College is an independent K-12 boys’ school in Sydney, Australia. Students come from affluent socio-economic backgrounds, and most live in highly developed suburban environments. Enrolment is not based on academic performance; however, a small number of students receive academic scholarships. The Glengarry campus of the school is located in a bush environment about 3 h’ drive away from the main school, and facilities are comprised of simple dormitory accommodation blocks, classrooms, a dining hall and recreational spaces.

The Glengarry program has been running continuously since 1989, and is compulsory for all students in Year 9 (ages 14–15). There are approximately 200 students in each year, and they are assigned to a first or second semester intake to the Glengarry program. Students spend two school terms (about 20 weeks) away from their families at the Glengarry campus, living in dormitories of about 20 boys, participating in regular school classes for 5 days of the week, undertaking regular running and mountain biking activities before and after school, and going on outdoor adventure trips over the remaining 2 days of the week. Peak outdoor adventures occur at the end of the first term (e.g., 4-day hike, 6-h rogaine orienteering competition) and at the culmination of the program (e.g., 3-day solo, 24-h rogaine competition, 6-day “Long Journey Home” hike and bike trip back to the main school). The Glengarry program thus incorporates the outdoor learning forms of OAE through the formal outdoor adventure trips, and LOTC to the extent that teachers choose to take their classes outdoors. The residential nature of the program is also an important element of the program, facilitating development of relationships with peers and teachers.

### Study design

The 2019 cohort of Year 9 Glengarry students were followed before, during and after their two-term outdoor learning program, and the experience of Intake 1 students was compared with a waitlist group (Intake 2) who undertook the Glengarry program 6 months later. Engagement and 21st Century skills surveys were administered to students at key time points, with respondent number for each survey detailed in Table 1. Practical constraints prevented survey 2 being administered to both intakes simultaneously, with Intake 1 unavailable in June 2019 while away on peak adventure trips.

**Table 1:** Quantitative survey administration time points and respondent numbers.

Time point	Intake 1	Respondents	Intake 2	Respondents
1	February 2019 (1 month into Glengarry)	104	February 2019 (5 months before Glengarry)	78
2	August 2019 (2 months after Glengarry)	49	June 2019 (1 month before Glengarry)	75
3	February 2020 (8 months after Glengarry)	82	February 2020 (2 months after Glengarry)	74
4			October 2020 (10 months after Glengarry)	40

A focus group was conducted with six students from each intake in the month after they completed their final quantitative survey. Purposive sampling was used to randomly select three students from those with high quantitative engagement scores and three from those with low scores from each intake. The focus groups followed a semi-structured design utilizing pre-determined questions around the key themes of engagement and 21st Century skills, and also with capacity to follow matters raised by the students.

This mixed method design enabled a quantitative exploration of student-rated student engagement and 21st Century skills over time, as well as a qualitative student reflection on the contributing factors behind these changes. Creswell (2015) defined this approach as an explanatory sequential design, where quantitative measures are first used to identify the breadth of an effect and then qualitative methods help to explain these results in more depth.

### Participants

The first semester Glengarry intake of 2019 was comprised of 108 boys, who participated in the program between February and June with a 2-week school holiday break in April. The second semester intake of 102 boys undertook regular classes at the main school during the first half of 2019, and could be regarded as a “waitlist” group before participating in the Glengarry program between July and December (with a 2-week school holiday break in October). Rather than the school allocating students to either intake using any single factor (e.g., academic ability, effort grades, or sport team), consideration was given to achieving a broad mix of students in each intake. A small number of students were allocated to a particular intake to fit in with elite sporting commitments. The average student age in February 2019 was 14 years and 6 months for Intake 1, and 14 years and 5 months for Intake 2.

Because the Glengarry program is compulsory for all Year 9 students, participation in the research study did not involve any additional intervention except completing surveys at four time points across 2 years. Students and their parents were informed in writing about the study requirements and given opportunity to opt out before the study began, and students were given this same opportunity to opt out at the start of every survey. Time was allocated during pastoral lessons to complete each survey. No students were excluded from participation in the study. Survey data was de-identified by usage of an identification number to link student data across surveys. Purposive sampling was used to identify candidates for a focus group of six students from each intake, representing high and low levels of engagement. Participation in a focus group was voluntary, and some students declined and were replaced from the high or low engagement list. Ethics approval for the study was obtained from the University of Western Sydney (H13009).

### Instruments

#### Motivation and engagement scale

The Motivation and Engagement Scale (MES) is an Australian instrument originally developed by Martin (2007) and validated across a range of student contexts and age groups. In psychometric testing with over 12,000 students across 38 Australian high schools, the MES instrument has shown within and between network validity, internal consistency (Cronbach alpha coefficients over 0.7), and invariance across gender and school year levels (Martin, 2007). The MES has since been utilized in university, workplace, music and sport settings (Martin, 2008b), and across the academic lifespan (Martin, 2009). The MES contains 44 items representing 11 self-reported dimensions, including six motivation factors (learning focus, valuing, self-belief, anxiety, failure avoidance, uncertain control) and five engagement factors (planning and monitoring, task management, persistence, disengagement, self-sabotage).

#### 21st Century skills scale

In the absence of a psychometrically validated instrument in the academic literature which measures self-reported 21st Century skills, a 16 item 21st Century Skills Scale (21CSS) was developed based on the four core capabilities (communication, collaboration, creativity, critical thinking) proposed by Fadel (2016) and present in most 21st Century skills frameworks (refer to Introduction). Four statements were written for each skill area, synthesized from the Center for Curriculum Redesign (2019) subcategories, with seven point Likert-style response options for consistency with the MES. The prototype 21CSS instrument was piloted with 71 participants in the case study school, and the wording of one item was adjusted based on their feedback of understanding the questions. Table 2 shows that correlations between items within each skill area were in the moderate range (0.3–0.59) for the prototype instrument, according to Cohen (1988). In terms of reliability, the 21CSS instrument had an overall Cronbach Alpha value of 0.88, and individual skills were above 0.6 (see Table 2). Pallant (2010) recommended that Cronbach alpha coefficients should ideally be above 0.7, however noted that the statistic is sensitive to the number of items in a scale and that coefficients can be lower in shorter scales. The baseline administration of the final 21CSS survey revealed similar moderate inter-item correlations for each intake group respectively: creativity (0.25, 0.35), critical thinking (0.38, 0.37), communication (0.40, 0.45) and collaboration (0.39, 0.29). Based on these correlations, the 21CSS was considered to have sufficient validity as a self-report measure of the four identified 21^st^ Century skills.

**Table 2:** 21st Century Skills Scale correlations and reliability.

21st century skill	Mean inter-item correlation (Pearson’s co-efficient)	Reliability (Cronbach’s alpha)
Creativity	0.34	0.67
Critical thinking	0.37	0.71
Communication	0.38	0.71
Collaboration	0.32	0.64

## Results

The findings of this study are arranged into quantitative sections which respond to the first two research questions, and a qualitative section which addresses research question three.

### Quantitative findings

IBM SPSS Statistics (Version 27) was used for statistical analysis of quantitative data. Survey data points were arranged into a row for each student, which allowed analysis of within-subject comparison over time. Survey response rates were fairly consistent across time points, apart from a dip in Intake 1 responses at time point 2 and Intake 2 responses at time point 4 (Table 1). A simple *t*-test strategy for statistical analysis was used to compare the same group over two time points (within subjects) or the two intake groups across the same time point (between subjects). Statistics for each t-test are based on the cases with no missing or out-of-range data for any variable in the analysis. Prior to t-tests being undertaken, a histogram was produced for each variable at each time point to check for normal distribution.

#### Quantitative data from intake 1

**Figure 2:**
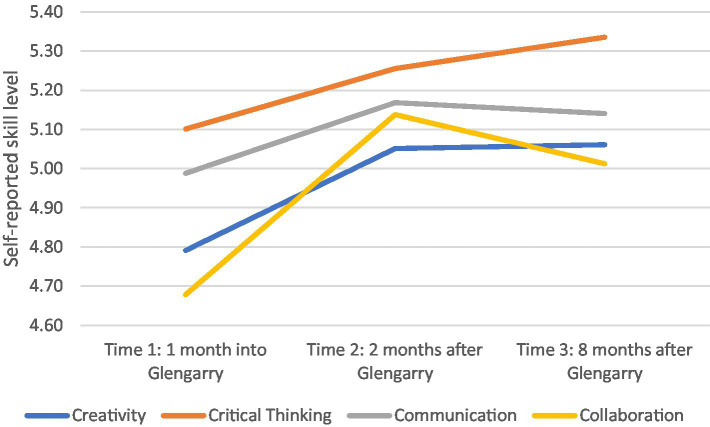
Intake 1 self-reported 21st Century skills at various time points.

The first two research questions concerned the quantitative changes in self-reported student motivation and engagement and 21st Century capabilities during and after the Glengarry extended residential and outdoor program. In summary, most 21st Century skills increased after the Glengarry program, and then remained static over the following 6 months back at school (refer to Figure 2).

**Figure 3:**
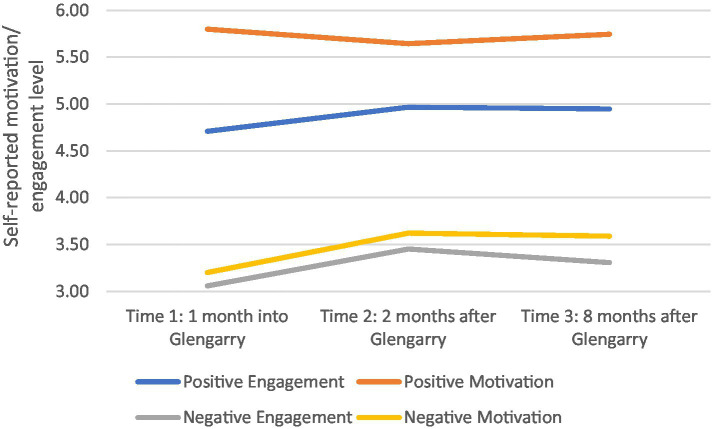
Intake 1 self-reported engagement and motivation at various time points.

In contrast, Figure 3 shows that positive motivation decreased and negative motivation and engagement factors increased once students returned to school, and similarly were unchanged over the next 6 months (except for positive motivation which recovered to its previous level).

Table 3 shows these changes in more detail with statistically significant differences at the *p* < 0.05 level indicated in bold. Between the start of the Glengarry program and 1 month afterwards (Time 1–2), Intake 1 students significantly increased in self-rated creativity, communication and collaboration, although perceived levels of critical thinking were unchanged. In contrast, once students had been back at school for 2 months, they felt significantly less positive motivation, including focus on learning (*p* = 0.027), and more negative motivation specifically focused on avoiding failure (*p* = 0.026). Positive engagement skills were unchanged after the Glengarry program, however students reported significantly more negative engagement strategies including disengagement (*p* = 0.021) and self-sabotage (*p* = 0.001).

**Table 3:** Intake 1 quantitative changes over time (significant changes at *p* < 0.05 level in bold).

	Creativity	Critical thinking	Communication	Collaboration	Positive engagement	Positive motivation	Negative engagement	Negative motivation
Time 1–2	**Increase *p* = 0.000**	No change *p* = 0.416	**Increase *p* = 0.046**	**Increase *p* = 0.007**	No change *p* = 0.118	**Decrease *p* = 0.018**	**Increase *p* = 0.005**	**Increase *p* = 0.018**
Time 2–3	No change *p* = 0.869	No change *p* = 0.333	No change *p* = 0.679	No change *p* = 0.640	No change *p* = 0.595	**Increase *p* = 0.023**	No change *p* = 0.724	No change *p* = 0.595
Time 1–3	**Increase *p* = 0.023**	**Increase *p* = 0.028**	No change *p* = 0.224	**Increase *p* = 0.003**	**Increase *p* = 0.049**	No *p* = 0.494	**Increase *p* = 0.030**	**Increase *p* = 0.002**

Between 1 and 8 months in their normal school routine after Glengarry (Time 2–3), the levels in self-rated 21st Century skills of Intake 1 students had plateaued, and there was no significant change in any of the four skill areas. In terms of positive motivation, students felt significantly more motivated about their interest in learning (*p* = 0.008) and ability to do well at school (*p* = 0.002), however their level of negative motivation (anxiety and control over their performance), their positive engagement skills (e.g., persistence, planning and time management), and the level of self-sabotage and disengagement remained unchanged.

Finally, when looking from the start of their Glengarry experience to 8 months afterwards (Time 1–3), Intake 1 students reported a significant increase in all 21st Century skill areas apart from communication. In other words, their improvement in creativity and collaboration after Glengarry endured, and while critical thinking did not significantly change over the first two time periods (Time 1–2 and Time 2–3) there was an overall significant increase from the start of Glengarry to 8 months afterward (Time 1–3). The initial significant increase in communication skills during Glengarry (Time 1–2) then underwent a slight (but not significant) decrease in the post-Glengarry (Time 2–3) period, resulting in a non-significant increase over the whole study (Time 1–3). There was an overall significant rise in negative engagement and negative motivation factors upon the students return to school, and these stayed similarly depressed over the next 7 months (Time 2–3) resulting in an overall decrease across the study (Time 1–3).

Positive motivation was unchanged over the duration of the whole study (Time 1–3), after an initial drop (Time 1–2) and subsequent rise (Time 2–3). Positive engagement (especially planning skills; *p* = 0.019) significantly increased over the whole measurement period (Time 1–3) even though there were no significant changes in the first two-time intervals (Time 1–2 and Time 2–3).

#### Supporting quantitative data from intake 2

During the same 6-month period that Intake 1 students went through the Glengarry program, Table 4 shows that Intake 2 students (of very similar age and maturation) experiencing regular schooling did not record any significant changes in 21st Century skills or (positive or negative) engagement and motivation factors (Time 1–2).

**Table 4:** Intake 2 quantitative changes over time (significant changes at *p* < 0.05 level in bold).

	Creativity	Critical thinking	Communication	Collaboration	Positive engagement	Positive motivation	Negative engagement	Negative motivation
Time 1–2	No change *p* = 0.739	No change *p* = 0.266	No change *p* = 0.282	No change *p* = 0.295	No change *p* = 0.102	No change *p* = 0.501	No change *p* = 0.541	No change *p* = 0.928
Time 1–3	No change *p* = 0.525	No change *p* = 0.312	No change *p* = 0.613	No change *p* = 0.746	No change *p* = 0.079	**Decrease** *p* = 0.024	No change *p* = 0.637	No change *p* = 0.189
Time 3–4	No change *p* = 0.385	No change *p* = 0.730	No change *p* = 0.956	No change *p* = 0.736	No change *p* = 0.885	No change *p* = 0.812	No change *p* = 0.500	No change *p* = 0.344

Similarly to Intake 1, the positive motivation of Intake 2 students was significantly lower after Glengarry than 6 months beforehand [and specifically the subscales of learning focus (*p* = 0.032) and self-belief (*p* = 0.036)] and there was no change in positive engagement skills (Time 1–3). Intake 2 did not show the same statistically significant increases in negative motivation or engagement after their return to school as did Intake 1 (Time 1–3), however there was an increase in these means (0.25 and 0.15 respectively) indicating a similar trend of lower motivation and engagement. Intake 2 students did not report any significant change in 21st Century skills from 5 months before Glengarry to 2 months afterward (Time 1–3). Between 2 and 10 months of being back at school, Intake 2 students followed a similar pattern to Intake 1 of no significant differences in outcome variables (Time 3–4).

In summary, while Intake 2 students did not report significant gains in 21st Century skills from 5 months before Glengarry to 2 months afterward, small decreases in motivation and no change in positive engagement after Glengarry were similar to Intake 1. The levels of 21st Century skills, motivation and engagement reported by Intake 2 students did not change in the subsequent 8 months of school, matching Intake 1 results for the same post-program period.

T-tests comparing the two student groups at the same time relative to their Glengarry experience (Table 5) showed that Intake 2 students reported significantly higher 21st Century skills and positive engagement before the program, whereas there was no difference between intake groups in three of the four motivation and engagement factors. Even though Intake 2 had higher reported baseline levels of 21st Century skills, there were no significant differences between intakes both 2 months and 8–10 months after their Glengarry experience.

**Table 5:** Differences between Intakes at similar durations before and after Glengarry (significant changes at *p* < 0.05 level in bold).

	Creativity	Critical thinking	Communication	Collaboration	Positive engagement	Positive motivation	Negative engagement	Negative motivation
1 month before/into Glengarry	**Yes *p* = 0.025**	**Yes *p* = 0.013**	**Yes *p* = 0.005**	**Yes *p* < 0.001**	**Yes *p* = 0.015**	No *p* = 0.970	No *p* = 0.713	No *p* = 0.707
2 months after Glengarry	No *p* = 0.401	No *p* = 0.922	No *p* = 0.733	No *p* = 0.881	No *p* = 0.096	No *p* = 0.689	No *p* = 0.254	No *p* = 0.676
8–10 months after Glengarry	No *p* = 0.944	No *p* = 0.406	No *p* = 0.242	No *p* = 0.320	No *p* = 0.082	No *p* = 0.890	No *p* = 0.380	No *p* = 0.333

#### Summary of quantitative results

The first research question in this study concerned whether the Glengarry residential and outdoor program had an effect on the target variables of motivation, engagement and 21st Century skills. The quantitative data from Intake 1 clearly supported the hypothesis that 21st Century skills increased during the program, and the lack of significant changes for Intake 2 across the same time period confirmed that this change was not simply due to maturational development. Intake 2 students did not report significant gains in 21st Century skills across the program period, however their pre-program levels were high compared to those of Intake 1.

The second research question enquired whether these increases at Glengarry were enduring after return to the normal school environment, and the quantitative data suggested a more complicated pattern of change. Three of the four 21st Century capabilities increased during the Glengarry program for Intake 1 students and remained at these elevated levels for the next 8–10 months in the standard school setting, and while the increase of the fourth skill area (communication) did not achieve statistical significance across the Glengarry program it was significantly higher 8 months afterwards. In contrast, while positive engagement was unchanged for both intakes after 2 months back at school, there were significant decreases in positive motivation (both intakes) and increased negative engagement and motivation (Intake 1). In the following 6–8 months at school, the dip in positive motivation reported by Intake 1 students returned to its original level, however there were no changes to other 21st Century skills and engagement and motivation factors for both intake groups.

### Qualitative insights

Research question three sought to explore the contributing factors to the quantitative changes recorded above, by means of semi-structured focus groups with students from each Glengarry intake. The two focus groups were conducted with a representative sample of students with low and high engagement, and provide deeper understanding of the quantitative results through hearing student voice. Two collaborators (the first author and an independent education researcher) were provided with a list of the *a-priori* factors of motivation, engagement and 21st Century skills, and separately coded the focus group transcripts. The two first round code lists were then consolidated into a master code list, and the same collaborators again coded the transcripts independently using this master list. Discrepancies in second round coding decisions were discussed, and coding adjusted in some cases, resulting in an overall Kappa coefficient of 0.87. Table 6 shows an excellent (>0.75) level of agreement for all codes except collaboration and agency in learning, which were both in the higher end of the good (0.40–0.75) agreement range. The 12 codes were arranged into four themes, described below.

**Table 6:** Qualitative codes and themes, with Kappa coefficient of inter-rater agreement.

Theme	Code	Kappa coefficient
Social capacities	Communication	0.85
	Collaboration	0.71
Cognitive capacities	Creative thinking	0.91
	Critical thinking	0.89
Engagement	Agency in learning	0.68
	Connection with natural environment	0.89
	Connection with peers	0.91
	Connection with teachers	0.88
	Enjoyment of learning	0.94
	Pressure in learning	0.95
Personal change	Personal development	0.90
	Transfer of learning	0.93

#### Theme 1: Social capacities

Students frequently mentioned the social capacities of communication (39 references) and collaboration (39 references). The experience of living closely together was positive for most students, but importantly they thought it necessitated the development of social capabilities. One student described *“you had to learn, um, like, how to interact with people that you do not choose to be around.”* This included dealing with conflict, as it was not possible to get away from peers at the end of the day like at school—*“with your dorm mates, instead of just, um, like, conflicting with someone, you could actually, like, talk it out maybe. And just, um, understand where everyone is coming from, because everyone is coming from different places in life.”* Outdoor activities also provided opportunities to develop social aptitudes, *“for example rock climbing, you have got two people down the bottom ensuring that you are not falling from a bloody wall, and so obviously you need to communicate between them how to be responsible.”* Collaboration and communication were regarded by students to be less important in normal school life, as classroom learning was perceived to have more of an individual focus. One student described: *“Once you go back to school, um you do not get as many opportunities to work together, and especially, like, going from Glengarry with 100 people back to a school with 1,000 people—um, it’s a lot more individual based, not community based, because it’s so much larger.”*

#### Theme 2: Cognitive capacities

Cognitive capacities were referenced slightly less frequently (20 references for creative thinking and 25 for critical thinking) but were still a major theme for students. There were mixed opinions as to the extent that their Glengarry experience developed creativity, however students agreed that hikes and solo camping facilitated creative thinking, at least in part because there was time to reflect: *“on hikes, there’s just a lot of time by yourself, just thinking, and then you get to really be creative about how you spend that time. For example, like, thinking about some possible, a novel or something. And there’s, just, like, a lot of time to think about that compared with at school.”* Students suggested that adventure activities with open-ended challenges promoted critical thinking skills, including hikes, rogaine, solo, and even putting up tents. *“The environment they put you in is quite natural – it’s not really something you can just be given a textbook to read about. They just, like, put you in situations, and, well I guess you think and adapt to what happens.”* Students perceived that there is opportunity to be creative at school across most school subjects, and particularly the humanities. Critical thinking was said to be useful in everyday life, and could be applied to strategies for academic study habits.

#### Theme 3: Engagement

The most prominent factors which made Glengarry engaging to students were the close connections with peers (48 references) and teachers (36 references). Although the social environment was intense, it built a rich sense of community which most students greatly valued: *“just being with those same people, like, in the same dorm,* [meeting] *at the flagpole, on hikes and through all of that, um, like, it was thick with real community, that you just cannot get back here.”* Students reported that this resulted in growth of social skills, conflict management strategies, self-confidence and increased ability to make new friends. Connection with teachers at Glengarry was no less significant, as one student described: *“I feel like I felt a lot more connected because again like, you are a lot more, like, emotionally connected with the teachers, and you are kind of like friends with them, and it is just so much easier to have a conversation with them.”* Seeing the teachers across different parts of Glengarry life contributed to this deeper sense of connection—*“what happened outside the classroom, like, made the relationship stronger between teachers and students”*—and the inverse was true back at school: *“Up here in Bellevue Hill* [main school campus]*, it’s a bit harder because we just see the teachers in the classroom and then we might see them around school but that’s about it.”* There was also a perception that teachers were less directive at Glengarry, which allowed greater student agency in learning: *“they did not really focus too much on teaching – they just answered your questions.”*

Connection with the natural environment was an unexpected finding, with 30 references across the two focus groups even though the topic was not raised by the interviewer. Students appreciated the opportunity to live in a natural environment and felt more connected to nature, as one student described—*“I think I just loved the whole outdoor experience, like, it’s pretty surreal.”* When it came to their academic work, boys enjoyed hands-on learning in the outdoors and preferred this even to using screens inside the classroom. Some students expressed that outdoor learning was not only a fun experience but also more effective for learning—*“my science teacher, often he’d take us outside to learn, and actually I enjoyed it a lot more, because I felt like I was getting more out of it. And it was just better, nicer, being out, like, with nature.”* One student summarized: *“to round up everything we said, I reckon we should do more outdoor activities and more, kind of, nature-based things. Like, not sitting in classrooms on laptops and stuff.”*

According to students, the learning culture at Glengarry was more relaxed and less stressful (27 references). One commented that *“at Glengarry it’s more laid back, and you have a lot of time”* and another replied *“Yeah, school at Glengarry was like a lot less formal.”* A third student was quick to point out that this relaxed environment wasn’t because little schoolwork was undertaken: *“I would not say that it is easier. It’s a lot different, like there is different components of it that, like, I have never done…we studied 4 texts in one term you know.”* Students described that the informal culture was influenced by: no homework, self-driven learning, more time, less formal classroom norms, no uniform requirement, closer relationship with teachers, and less rules. They categorically saw subsequent school life as more pressured, however were fatalistic about school becoming more stressful in senior years—*“going from Year 8, and then Year 9 and 10, and then into Year 11 and 12, it’s kind of like a really big ramp up in, like, the stress, and how much you have got to learn, and so I think in Year 9 going to Glengarry gives you like a break before, like the calm before the storm.”*

#### Theme 4: Personal change

The last identified theme was personal change, with personal development and transfer of learning codes both referenced frequently in the focus groups (33 and 34 references respectively). Students described understanding themselves and others better at Glengarry, and appreciating opportunities beyond school life. One student reflected: *“I learnt a lot about different people. And so, understood what, how people thought, how they acted, a lot more, and throughout Glengarry that I, like, shifted how I treated them, kind of, like, how they acted as well, and I kind just understood them a bit more, I guess.”* Students talked about growing in maturity, and specifically: self-management skills, self-confidence, good habits, and healthy diet. For example: *“I do not know if it was just ‘cause I got older, but I feel, down at Glengarry, I became more mature, and like, I feel like with maturity you get a better understanding of what’s important – what you find important.”* The school holidays provided a break after the intensity of Glengarry, and some students made personal changes during this time. Others described that it took them some time to get used to the routines of school: *“over the summer holidays I think we just tried to put our minds to rest. And then when we came back here, we were pretty slow.”*

In terms of transferring their learning from Glengarry to subsequent school life, students mentioned maintaining a greater confidence in learning, the ability to make new friends, and a healthier diet. One clearly articulated the connection between learning from a navigational adventure activity and the application of this learning in an academic context: *“with the rogaine, we have to plan out the best possible path. And like up here with studying you probably also have to plan the best possible way of studying.”* Not all gains made at Glengarry were transferred back into normal school life, as some students considered that the 21st Century skills they had learned at Glengarry were not needed as much at school. While a number of students felt that they could approach teachers for help more confidently, others thought that they were not able to ask questions as easily to teachers back at school. *“Like up here, it would be a lot harder to say like, oh sir, could you help me, and maybe after school down there they would help you out with something as well, but here you cannot really do that.”* In general, however, students considered that the skills they developed at Glengarry were useful for their subsequent school life: *“I think Glengarry helps create those skills, and then, coming back to Bellevue Hill, that’s just ensuring those skills are there. And so, you know, I think it’s important that we take these things from Glengarry, but ensure that they are all relevant to how we spend our last years at Bellevue Hill.”*

#### Summary of qualitative findings

Research question 3 aimed to explore students’ insights about the factors behind changes in 21st Century skills, motivation and engagement levels. Adventurous activities and an intense social environment were seen to drive the social capacities of communication and collaboration, whereas normal schooling was regarded to be more individually focused. The presence of open-ended situations in OAE was regarded to develop critical thinking skills, and space to think (for example while hiking) helped to build creativity.

The close connection with both peers and teachers at Glengarry was the strongest contributing factor to student motivation and engagement, including opportunities to interact with teachers in different contexts outside the classroom environment. Students found the more informal classroom culture engaging, and contrasted this with an expected ramp up in stress during their senior high school years. A closer connection with the natural environment was an unexpected driver of student engagement, and was even preferred to learning with digital devices which are typically enticing for boys.

Students considered that they had grown in maturity over the Glengarry program, and described development in self-awareness and understanding of others. Students generally felt that they could transfer skills learnt at Glengarry into their subsequent school life as needed, however were less confident about transferring their relational skills to teachers whom they only saw for shorter periods in the normal classroom context.

## Discussion

Both quantitative and qualitative data support the hypothesis that the Glengarry program boosted 21st Century skills and student motivation, however while students’ estimations of their personal skills remained high their motivation decreased on return to school. The immediate post-program levels of 21st Century skills, motivation and engagement generally endured over the next 8–10 months at school, both for Intake 1 students as they completed Year 9 (while Intake 2 was at Glengarry), and for both intakes back at school together in Year 10. These results are discussed in terms of the outcome variables, limitations of the study and recommendations for future research.

### 21st Century skills

Both in Australia (Lambert, 2017) and internationally (Organisation for Economic Cooperation Development, 2012), 21st Century skills have been recognized as important educational outcomes. However, there has been little policy advice or research evidence on how to effectively grow these capabilities (Lamb et al., 2017). This study demonstrates that challenging and open-ended OAE experiences in a rich social context builds the 21st Century skills of communication, collaboration, creativity and critical thinking, and that these gains can be maintained over time on return to the traditional school environment. It is acknowledged that the quantitative data across the program period did not show the same significant increase in 21st Century skills for Intake 2 students (Table 4) as for Intake 1 (Table 2), however comparison of the two groups prior to the program revealed that Intake 2 already had high self-reported levels of these capabilities (Table 5). Moreover, after Intake 1 gains across the Glengarry program, the two groups then had no statistical difference in their 21st Century skill levels 2 and 8–10 months later (Table 5). Even though there was no statistically significant increase in Intake 2 levels of 21st Century skills, students from this intake qualitatively described situations at Glengarry which had facilitated growth of all four 21st Century skills.

The only 21st Century competency which did not show a quantitatively significant improvement for Intake 1 after the Glengarry program was critical thinking, and yet students described in focus groups how various open-ended challenges and risk decisions at Glengarry required this 21st Century skill. Interestingly, students in the focus groups were less effusive about the development of creativity than critical thinking skills during Glengarry, whereas the quantitative data showed a significant increase in the former 1 month after the program but not the latter. There is only minimal evidence in the research literature for OAE improving critical and creative thinking, however this is due to a paucity of research with these outcome variables rather than findings of no change (Mann et al., 2022a). While a change in critical thinking may not have been sufficiently large to achieve statistical significance straight after Glengarry, there was a significant increase in perceived critical thinking between the start of Glengarry and 8 months afterward. As one student put it: *“I think critical thinking continually develops – it does not just, like, go up at Glengarry and just, I do not know, flatline. It’s something you, like, keep on building, like, through experience throughout your life.”* Perceived communication skills were significantly higher after Glengarry (Table 3) but then dipped slightly in the following 8 months at school, causing a non-significant change between Time 1–3. These quantitative changes match the students’ qualitative perception that communication skills were vital at Glengarry but not as important to individually-focused achievement at school.

### Motivation and engagement

Student motivation and engagement followed a different pattern over time to 21st Century skills, in that these factors generally decreased once students returned to school. Students’ sense of their positive engagement skills (planning, task management, and persistence) was largely unchanged; however, they were prone to feel less positively motivated and more detached from school while investing minimal effort in their school work (negative motivation). Intake 2 students did not record the same statistically significant deterioration in most motivation and engagement factors, however means trended in the same direction as Intake 1 showing an initial decrease after return to the normal school environment. Focus group comments indicated that students were able to internalize personal skills developed by Glengarry-specific experiences and could apply them to other environments, which is line with previous OAE research (e.g., Bobilya et al., 2015; Beames et al., 2020). In contrast, students’ sense of connection to school and learning seemed to be linked to the context they were in, and therefore changed once they returned to the main school environment. While there has been some research exploring the effect of regular short outdoor learning experiences on student motivation and engagement (e.g., Ruiz-Gallardo et al., 2013; Bølling et al., 2018), Richmond et al. (2018) noted that there is surprisingly little research on the effect of OAE experiences for student learning outcomes. Their study in an all-girls independent school in the United States found that annual multi-day OAE programs developed emotional engagement with school seen through increased rapport between students and with teachers. Additional research is required to explore whether the high level of engagement and motivation on OAE programs can be transferred back to the traditional school environment, and under what conditions.

Intake 1 students returned to traditional school learning for the second half of Year 9 while Intake 2 completed the Glengarry program, then the whole year group was back at school for the start of Year 10. It is possible that Intake 1 students showed no change in motivation, engagement and 21st Century skills over the first half year at school (between Time 2–3) because they were in limbo while they waited for the rest of the students to begin Year 10 together, but then may have experienced an uptick in the target variables once Year 10 was underway. The last measurement of Intake 2 students provides some clarification of this hypothesis, as it would show change between February and October of Year 10 (Time 3–4), however there was no significant change in 21st Century skills, engagement nor motivation across this period. These findings support the Intake 1 data which shows a lasting improvement in 21st Century skills, and a rise in motivation and engagement during Glengarry followed by a dip on return to the normal school environment.

Motivation and engagement were conceptualized by Martin (2007) as two inter-related constructs, yet students in the current study rated them differently after their Glengarry experience. In Martin’s model, positive engagement is linked to student skills (e.g., planning, task management, persistence) and these did not change after Glengarry. Negative engagement, on the other hand, describes how disengaged a student feels toward school, which was more prominent on return to the normal school environment. Positive motivation describes how a student sees themselves as a learner (e.g., self-belief, value placed on learning), and this recovered after an initial drop. Negative motivation rose after Glengarry and stayed elevated over the next 8 months, indicating that students were more anxious and felt less control in their normal school environment even though they had experienced a higher locus of control at Glengarry. In summary, students perceived that they developed learner capacities during their Glengarry experience, but upon return to the normal school context they felt less motivated about why they were at school and its relevance to them. One student described how he felt about school almost a year after Glengarry: *“My connection to school is just, I do not know, like, I come in and, like, go to school, like, learn some stuff, like, have fun with friends, and just go back home.”*

Some teachers at the case study school have anecdotally expressed that the Glengarry experience is unhelpful for students because a number come back less motivated than beforehand, which is line with the quantitative findings of this study. However, it is argued the spotlight should be on factors in the school environment that are demotivating for students, rather than removing the outdoor learning experience which raised their motivation. Students described that the significant motivational factors at Glengarry included their close relationship with peers and teachers, an increased connection to the natural environment, and a less formal classroom environment. Future research should explore how these factors can be incorporated into the normal school context, in line with literature on the importance of social facilitators of engagement (e.g., Furlong and Christenson, 2008; Lietaert et al., 2014) and international evidence on the benefits of outdoor learning (Mann et al., 2022b). Additionally, further research could investigate intentional strategies to maximize transfer of learning from OAE environments to the normal school context (Bolick et al., 2022).

### Limitations

A noteworthy strength of this study design was the benefit of intervention and waitlist groups which were matched in age, sex, and socio-economic status, in contrast to the OAE literature which sometimes lacks rigor in research design (Mann et al., 2022b). For example, Intake 1 showed gains over the outdoor program period (Table 3, Time 1–2) while Intake 2 did not show any significant changes over the same time period at school (Table 4, Time 1–2). Having said this, responses were much lower at two points which raises concerns over the validity of quantitative results at these time points (Table 1). Although the Intake 1 response rate was lower immediately after Glengarry, there were no significant differences in any target variables between the two intakes on their return to school (Table 5) and almost all Intake 1 significant changes from the Glengarry program endured across the next 8 months to time point 3 (Table 3) which had a higher response rate, indicating that time point 2 results were representative despite the lower number of responses. Intake 2 showed a similar drop in responses at time point 4 (10 months after Glengarry), and the validity of this data can be similarly be supported by the lack of any significant differences within Intake 2 between time points 3–4 and between the intakes 8–10 months after their Glengarry experience (Table 4).

A potential confounding factor in this study design was the two intakes had different levels of 21st Century skills and/or motivation and engagement from the start, even though they were not intentionally selected on the basis of these factors. Table 5 shows that Intake 2 students did indeed perceive their 21st Century skills and positive engagement to be significantly higher than Intake 1 immediately before the Glengarry program, which offers an explanation for why they did not significantly increase across the program period. Although Intake 1 students started with lower self-reported 21st Century skill levels, these rose across the program period to match Intake 2 such that there were no significant differences between the student groups either 2 or 8–10 months afterwards. Moreover, Intake 2 quantitative data showed the same trend (although not reaching statistical significance) of a slump in motivation and engagement after their initial return to school. From these observations, it can be concluded that similar program effects were taking place for both student intakes even though their baseline characteristics varied.

The authors acknowledge the quantitative instrument used to measure 21st Century skills had not been fully psychometrically validated, and recommend that such a process be undertaken for future research. Both quantitative instruments relied on student self-perception of 21st Century skills and motivation/engagement, which has been recommended as an effective methodology (Willms, 2015) but also has some limitations. An observer (for example a teacher) may have noticed changes which were not apparent to the students themselves, or countered a student’s perception that they were changing in a particular area. Also, students’ benchmarks may have increased as they got older, meaning that their self-expectations may have been higher in later measurement time points. This could potentially account for the decrease in student motivation; however, it would not explain that the students’ perceived engagement remained fairly static and 21st Century skills increased. Further research in this area could certainly benefit from a design which gathers and compares data from students, teachers and parents (McCourt and Griffiths, 2016). The construct validity of the MES has been demonstrated across various participant ages and contexts (Martin, 2008b, 2009). While the 21CSS is based on an established theoretical framework (Fadel, 2016), and the current study triangulated it with qualitative data sources, the 21CSS could undergo further construct validity testing as part of a comprehensive psychometric validation process (as suggested above).

## Conclusion

This study sought to provide evidence to respond to three research questions. The first was around whether the 6-month Glengarry residential and outdoor program increased Year 9 boys’ self-rated motivation, engagement and 21st Century skills, and both quantitative and qualitative data supported the benefit of the Glengarry program in all these factors. The second question asked whether these gains were maintained on the students’ return to their normal school context and for the next year, and the data painted a more complex picture of these changes. The gains in 21st Century capabilities were evident in the first month back at school, and endured over the next 8–10 months. Positive engagement skills (planning, task management, and persistence) remained stable in the year after Glengarry, but students’ motivation decreased on their initial return to school and then partially recovered after 8–10 months. These effects were not simply due to maturation, as they were not mirrored in the waitlist Intake 2 cohort who experienced a normal semester at school at the same time. Nor were they due to inherent differences between the two intakes, as Intake 1 started with lower levels but there were no significant differences between groups at both 2 and 8–10 months after Glengarry. The third question enquired about contributing factors to the gains in outcome variables, and qualitative data revealed that students perceived connections with peers, teachers and the natural environment were key environmental factors which boosted 21st Century skills and motivation at Glengarry.

The challenge arising out of this study is how these key factors can be incorporated into a standard high school experience for every student. Further research could explore how the connections with peers, teachers and the natural environment could be integrated into current models for schooling; which specific elements of OAE and LOTC effect student gains in engagement and 21st Century skills; how boosted engagement and motivation levels on outdoor learning programs can be transferred back to the traditional school context; and, whether these gains could be achieved on shorter outdoor learning programs.

## Data availability statement

The raw data supporting the conclusions of this article will be made available by the authors, without undue reservation.

## Ethics statement

The studies involving human participants were reviewed and approved by Western Sydney University (H13009). Written informed consent to participate in this study was provided by the participants’ legal guardian/next of kin.

## Author contributions

JM, TG, and ST contributed to the study design. JM collected and analyzed data and wrote the first draft of the manuscript. TG and ST helped to reshape the manuscript and expand the literature review. All authors contributed to the article and approved the submitted version.

## Conflict of interest

The authors declare that the research was conducted in the absence of any commercial or financial relationships that could be construed as a potential conflict of interest.

## Publisher’s note

All claims expressed in this article are solely those of the authors and do not necessarily represent those of their affiliated organizations, or those of the publisher, the editors and the reviewers. Any product that may be evaluated in this article, or claim that may be made by its manufacturer, is not guaranteed or endorsed by the publisher.

## References

[ref1] Abbott-Chapman J. Martin K. Ollington N. Venn A. Dwyer T. Gall S. (2014). The longitudinal association of childhood school engagement with adult educational and occupational achievement: findings from an Australian national study. Br. Educ. Res. J. 40, 102–120. doi: 10.1002/berj.3031

[ref2] Adams D. Gray T. (2023). An exploration of how the disruption of mainstream schooling during the COVID-19 crisis provided opportunities that we can learn from so that we may improve our future relationship with the more-than-human world. SN Soc. Sci. 3:18. doi: 10.1007/s43545-022-00588-1, PMID: 36686567 PMC9839208

[ref3] Anderson M. Jefferson M. (2018). Transforming organizations: Engaging the 4Cs for powerful organizational learning and Change. Sydney: Bloomsbury.

[ref4] Angus M. McDonald T. Ormond C. Rybarcyk R. Taylor A. Winterton A. (2009). The pipeline project: Trajectories of classroom behaviour and academic progress: A study of student engagement with learning. Mount Lawley, Western Australia: Edith Cowan University.

[ref5] Appleton J. J. Christenson S. L. Furlong M. J. (2008). Student engagement with school: critical conceptual and methodological issues of the construct. Psychol. Sch. 45, 369–386. doi: 10.1002/pits.20303

[ref6] Archambault I. Janosz M. Fallu J.-S. Pagani L. S. (2009). Student engagement and its relationship with early high school dropout. J. Adolesc. 32, 651–670. doi: 10.1016/j.adolescence.2008.06.007, PMID: 18708246

[ref7] Australian Curriculum Assessment and Reporting Authority. (2018). General capabilities in the Australian curriculum (Version 8.4). Available at: https://www.australiancurriculum.edu.au/f-10-curriculum/general-capabilities/

[ref8] Beames S. Higgins P. Nicol R. (2012). Learning outside the classroom: Theory and guidelines for practice. New York, NY: Routledge.

[ref9] Beames S. Mackie C. Scrutton R. (2020). Alumni perspectives on a boarding school outdoor education programme. J. Advent. Educ. Outdoor Learn. 20, 123–137. doi: 10.1080/14729679.2018.1557059

[ref10] Becker C. Lauterbach G. Spengler S. Dettweiler U. Mess F. (2017). Effects of regular classes in outdoor education settings: a systematic review on students’ learning, social and health dimensions. Int. J. Environ. Res. Public Health 14:485. doi: 10.3390/ijerph14050485, PMID: 28475167 PMC5451936

[ref11] Bobilya A. J. Kalisch K. Daniel B. Coulson E. R. (2015). An investigation of participants' intended and actual transfer of learning following an outward bound wilderness experience. J. Outdoor Recreat. Educ. Leadersh. 7, 93–111. doi: 10.18666/JOREL-2015-V7-I2-7006

[ref12] Bolick C. M. Glazier J. Stutts C. (2022). Taking off the backpacks: the transference of outdoor experiential education to the classroom. J. Outdoor Recreat. Educ. Leadersh. 14, 54–71. doi: 10.18666/JOREL-2022-V14-I2-11137

[ref13] Bølling M. Otte C. R. Elsborg P. Nielsen G. Bentsen P. (2018). The association between education outside the classroom and students’ school motivation: results from a one-school-year quasi-experiment. Int. J. Educ. Res. 89, 22–35. doi: 10.1016/j.ijer.2018.03.004

[ref14] Center for Curriculum Redesign. (2019). Competencies/subcompetencies framework. Available at: https://curriculumredesign.org/framework/

[ref15] Cohen J. (1988). Statistical power analysis for the behavioral sciences (2nd) Edn. Hillsdale, N.J: L. Erlbaum Associates.

[ref16] Creswell J. W. (2015). A concise introduction to mixed methods research. Thousand Oaks, California: SAGE.

[ref17] Fadel C. (2016). Redesigning the curriculum for a 21st century education. Available at: https://curriculumredesign.org/wp-content/uploads/CCR-FoundationalPaper-Updated-Jan2016.pdf

[ref18] Fraysier K. Reschly A. Appleton J. (2020). Predicting postsecondary enrollment with secondary student engagement data. J. Psychoeduc. Assess. 38, 882–899. doi: 10.1177/0734282920903168

[ref19] Fredricks J. Blumenfeld P. Paris A. (2004). School engagement: potential of the concept, state of the evidence. Rev. Educ. Res. 74, 59–109. doi: 10.3102/00346543074001059

[ref20] Fullan M. Langworthy M. (2013). *Towards a new end: New pedagogies for deep learning*. Seattle, Washington. Available at: https://michaelfullan.ca/wp-content/uploads/2013/08/New-Pedagogies-for-Deep-Learning-An-Invitation-to-Partner-2013-6-201.pdf

[ref21] Furlong M. J. Christenson S. L. (2008). Engaging students at school and with learning: a relevant construct for all students. Psychol. Sch. 45, 365–368. doi: 10.1002/pits.20302

[ref22] Gallup A. (2014). State of America’s schools: The path to winning again in education. Washington, DC: Gallup Inc.

[ref23] Goss P. Sonnemann J. Griffiths K. (2017). *Engaging students: creating classrooms that improve learning* (2017-01). Grattan Institute. Available at: https://grattan.edu.au/report/engaging-students-creating-classrooms-that-improve-learning/

[ref24] Gray T. (1997). *The impact of an extended stay outdoor education school program upon adolescent participants.* Doctor of philosophy thesis. University of Wollongong. Available at: http://ro.uow.edu.au/theses/1799

[ref25] Griffiths K. Webber A. (2017). *Tell them from me: gender and engagement*. Centre for Education Statistics and Evaluation. Available at: https://www.cese.nsw.gov.au/images/stories/PDF/TTFM_gender_and_engagement_F17_AA.pdf

[ref26] Hancock K. J. Zubrick S. (2015). *Children and young people at risk of disengagement from school*: Commissioner for children and young people. Western Australia: Commissioner for Children and Young People.

[ref27] Hattie J. Marsh H. W. Neill J. T. Richards G. E. (1997). Adventure education and outward bound: out-of-class experiences that make a lasting difference. Rev. Educ. Res. 67, 43–87. doi: 10.2307/1170619

[ref28] Klem A. M. Connell J. P. (2004). Relationships matter: linking teacher support to student engagement and achievement. J. Sch. Health 74, 262–273. doi: 10.1111/j.1746-1561.2004.tb08283.x, PMID: 15493703

[ref29] Kolb D. A. (1984). Experiential learning: Experience as the source of learning and development. Englewood Cliffs, N.J.: Prentice-Hall.

[ref30] Lamb S. Maire Q. Doecke E. (2017). *Key skills for the 21st century: An evidence-based review*. NSW Department of Education. Available at: https://education.nsw.gov.au/content/dam/main-education/teaching-and-learning/education-for-a-changing-world/media/documents/Key-Skills-for-the-21st-Century-Executive-Summary.pdf

[ref31] Lambert P. (2017). *Hard focus on “soft” skills*. Available at: https://education.nsw.gov.au/our-priorities/innovate-for-the-future/education-for-a-changing-world/media/documents/exar/Hard_focus_on_soft_skills_Dr_Phil_Lambert.pdf

[ref32] Lietaert S. Verschueren K. De Fraine B. Laevers F. (2014). Indicators and facilitators of student engagement in secondary education: reviewing the gender gap. In: *Paper presented at the international congress for school effectiveness and improvement (ICSEI) conference*, Yogyakarta, Indonesia.

[ref33] Maehr M. L. Meyer H. A. (1997). Understanding motivation and schooling: where We've been, where we are, and where we need to go. Educ. Psychol. Rev. 9, 371–409. doi: 10.1023/A:1024750807365

[ref34] Mann J. (2018). Is school working for teenage boys? Outdoor learning and real-life skills could be the keys to re-engagement. Curric. Pers. 38, 169–174. doi: 10.1007/s41297-018-0051-0

[ref35] Mann J. Gray T. Truong S. (2022a). Rediscovering the potential of outdoor learning for developing 21st century capabilities. In Jucker R. Au J. von (Eds.), High quality outdoor learning: Evidence-based education outside the classroom for children. Teachers and Society: Springer Nature.

[ref36] Mann J. Gray T. Truong S. Brymer E. Passy R. Ho S.. (2022b). Getting out of the classroom and into nature: a systematic review of nature-specific outdoor learning on school Children's learning and development. Front. Public Health 10:877058. doi: 10.3389/fpubh.2022.877058, PMID: 35651851 PMC9149177

[ref37] Mann J. Gray T. Truong S. Sahlberg P. Bentsen P. Passy R.. (2021). A systematic review protocol to identify the key benefits and efficacy of nature-based learning in outdoor educational settings. Int. J. Environ. Res. Public Health 18:1199. doi: 10.3390/ijerph18031199, PMID: 33572827 PMC7908363

[ref38] Martin A. J. (2005). Exploring the effects of a youth enrichment program on academic motivation and engagement. Soc. Psychol. Educ. 8, 179–206. doi: 10.1007/s11218-004-6487-0

[ref39] Martin A. J. (2007). Examining a multidimensional model of student motivation and engagement using a construct validation approach. Br. J. Educ. Psychol. 77, 413–440. doi: 10.1348/000709906X118036, PMID: 17504555

[ref40] Martin A. J. (2008a). Enhancing student motivation and engagement: the effects of a multidimensional intervention. Contemp. Educ. Psychol. 33, 239–269. doi: 10.1016/j.cedpsych.2006.11.003

[ref41] Martin A. J. (2008b). Motivation and engagement in diverse performance settings: testing their generality across school, university/college, work, sport, music, and daily life. J. Res. Pers. 42, 1607–1612. doi: 10.1016/j.jrp.2008.05.003

[ref42] Martin A. J. (2009). Motivation and engagement across the academic life span. Educ. Psychol. Meas. 69, 794–824. doi: 10.1177/0013164409332214

[ref43] Martin A. J. Martin T. G. Evans P. (2016). Motivation and engagement in Jamaica: testing a multidimensional framework among students in an emerging regional context. J. Psychoeduc. Assess. 36:0734282916674424. doi: 10.1177/0734282916674424

[ref44] Martino W. (2008). Boys' underachievement: Which boys are we talking about? Ontario: Literacy and Numeracy Secretariat.

[ref45] McCarthy I. McCourt B. (2017). Improving high school engagement, classroom practices and achievement. Sydney: Centre for Education Statistics and Evaluation.

[ref46] McCourt B. Griffiths K. (2016). Capturing and measuring student voice. Centre for Education Statistics and Evaluation. Available at: https://www.cese.nsw.gov.au/publications-filter/learning-curve-15-capturing-and-measuring-student-voice

[ref47] McCree M. Cutting R. Sherwin D. (2018). The hare and the tortoise go to Forest School: taking the scenic route to academic attainment via emotional wellbeing outdoors. Early Child Dev. Care 188, 980–996. doi: 10.1080/03004430.2018.1446430

[ref48] McLeod B. Allen-Craig S. (2007). What outcomes are we trying to achieve in our outdoor education programs? Aust. J. Outdoor Educ. 11, 41–49. doi: 10.1007/BF03400856

[ref49] Miller N. C. Kumar S. Pearce K. L. Baldock K. L. (2021). The outcomes of nature-based learning for primary school aged children: a systematic review of quantitative research. Environ. Educ. Res. 27, 1115–1140. doi: 10.1080/13504622.2021.1921117

[ref50] Mygind L. Kjeldsted E. Hartmeyer R. Mygind E. Bølling M. Bentsen P. (2019). Mental, physical and social health benefits of immersive nature-experience for children and adolescents: a systematic review and quality assessment of the evidence. Health Place 58:102136. doi: 10.1016/j.healthplace.2019.05.014, PMID: 31220797

[ref51] Nicol R. Waite S. (2020). “Outdoor learning” in Encyclopedia of teacher education. ed. Peters M. A. (Singapore: Springer), 1–6.

[ref52] OECD. (2019). OECD skills strategy 2019: Skills to shape a better future. Paris: OECD Publishing.

[ref53] Organisation for Economic Cooperation Development (2012). Better Skills, Better Jobs, Better Lives: A Strategic Approach to Skills Policies Organisation for Economic Cooperation Development.

[ref54] Organisation for Economic Cooperation Development (2013). PISA 2012 results: Ready to learn (volume III): Students' engagement, Drive and Self-Beliefs OECD Publishing.

[ref55] Organisation for Economic Cooperation Development. (2015). *The ABC of gender equality in education: Aptitude, behaviour, confidence* (9264230025).

[ref56] Pallant J. (2010). SPSS survival manual: A step by step guide to data analysis using SPSS (4th) Edn. Berkshire: McGraw-Hill Education.

[ref57] Pendergast D. (2022). Conversations that matter: Rethinking teaching and teacher tducation in a post pandemic world. Brisbane, Queensland: Griffith University.

[ref58] Price A. (2015). Improving school attendance: can participation in outdoor learning influence attendance for young people with social, emotional and behavioural difficulties? J. Advent. Educ. Outdoor Learn. 15, 110–122. doi: 10.1080/14729679.2013.850732

[ref59] Priest S. Gass M. (2005). Effective leadership in adventure programming (2nd). Champaign, Ill: Human Kinetics.

[ref60] Richmond D. Sibthorp J. Gookin J. Annarella S. Ferri S. (2018). Complementing classroom learning through outdoor adventure education: out-of-school-time experiences that make a difference. J. Advent. Educ. Outdoor Learn. 18, 36–52. doi: 10.1080/14729679.2017.1324313

[ref61] Ruiz-Gallardo J.-R. Verde A. Valdés A. (2013). Garden-based learning: an experience with "at risk" secondary education students. J. Environ. Educ. 44, 252–270. doi: 10.1080/00958964.2013.786669

[ref62] Ryan R. M. Deci E. L. (2000). Self-determination theory and the facilitation of intrinsic motivation, social development, and well-being. Am. Psychol. 55, 68–78. doi: 10.1037/0003-066X.55.1.68, PMID: 11392867

[ref63] Scoular C. Ramalingam D. Duckworth D. Heard J. (2020). *Assessment of general capabilities: Skills for the 21st century learner. Final Report*. Camberwell, VIC. Available at: https://research.acer.edu.au/ar_misc/47

[ref64] Sibthorp J. Furman N. Paisley K. Gookin J. Schumann S. (2011). Mechanisms of learning transfer in adventure education: qualitative results from the NOLS transfer survey. J. Exp. Educ. 34, 109–126. doi: 10.1177/105382591103400202

[ref65] Tomaszewski W. Xiang N. Western M. (2020). Student engagement as a mediator of the effects of socio-economic status on academic performance among secondary school students in Australia. Br. Educ. Res. J. 46, 610–630. doi: 10.1002/berj.3599

[ref66] Tramonte L. Willms J. (2012). “Anxiety and emotional discomfort in the school environment: The interplay of school processes, learning strategies, and children’s mental health,” in Public Health - Social and Behavioral Health. ed. Maddock J. (Rijeka, Croatia: InTech), 461–476.

[ref67] Trilling B. (2009). 21st century skills: Learning for life in our times (1st Edn.). San Francisco, CA: Jossey-Bass.

[ref68] White R. L. Bennie A. Vasconcellos D. Cinelli R. Hilland T. Owen K. B.. (2021). Self-determination theory in physical education: a systematic review of qualitative studies. Teach. Teach. Educ. 99:103247. doi: 10.1016/j.tate.2020.103247

[ref69] Willms J. D. (2015). *Student Engagement and Wellbeing in NSW*. NSW Education & Communities, Centre for Education Statistics and Evaluation. Available at: https://www.cese.nsw.gov.au/images/stories/PDF/LearningCurve7_TTFM_May2015.pdf

[ref70] Willms J. D. Frieson S. Milton P. (2009). What did you do in school today? Transforming classrooms through social, academic, and intellectual engagement. (First National Report). Toronto, Canada: Canadian Education Association. AVailable at: http://www.cea-ace.ca/sites/cea-ace.ca/files/cea-2009-wdydist.pdf

[ref71] World Economic Forum. (2015). New vision for education: Unlocking the potential of technology. Geneva, Switzerland. Available at: http://www3.weforum.org/docs/WEFUSA_NewVisionforEducation_Report2015.pdf

[ref72] Yazzie-Mintz E. (2006). Voices of students on engagement. A report on the 2006 high school survey of student Engagement. Bloomington, Indiana: Center for Evaluation and Education Policy.

